# Effects of commonly used chemical fertilizers on development of free-living stages of *Haemonchus contortus* in experimentally infected pasture

**DOI:** 10.14202/vetworld.2017.764-768

**Published:** 2017-07-10

**Authors:** Tapas Kumar Roul, Mitra Rajan Panda, Bijayendranath Mohanty, Kautuk Kumar Sardar, Manaswini Dehuri, Ananta Hembram, Trilochan Mohapatra

**Affiliations:** 1 Department of Veterinary Parasitology, College of Veterinary Science & Animal Husbandry, Orissa University of Agriculture & Technology, Bhubaneswar - 751 003, Odisha, India; 2 Department of Veterinary Pharmacology & Toxicology, College of Veterinary Science & Animal Husbandry, Orissa University of Agriculture & Technology, Bhubaneswar - 751 003, Odisha, India

**Keywords:** *Haemonchus contortus*, larva, N-P-K fertilizer, pasture

## Abstract

**Aim:**

The effects of N-P-K fertilizers in the form of urea, single super phosphate and muriate of potash on development of free-living stages of *Haemonchus contortus* were studied.

**Materials and Methods:**

Five parasite free experimental plots of 1 m×1 m area, each of paddy leaves (15-day-old) and an equal number of *Cynodon dactylon* grass were infested with about 10×10^4^ eggs/ml phosphate buffer saline along with the application of the calculated amount of fertilizers solution. On the 10^th^ day of posttreatment, the pasture was cut, processed, larvae recovered by Baermann method and counted, which was expressed as number of L_3_ per kg dry matter (DM) of pasture.

**Results:**

The average recovered population of L_3_ of *H. contortus* per kg DM varied significantly (p<0.05) between the paddy leaves (5933.57±22.718) and *Cynodon* grass (4861.00±22.718). When different doses of chemical fertilizer and their impact on different pasture were analyzed for control (T-1, 0-0-0 kg/ha N-P-K), the mean L_3_ recovery per kg DM of paddy (19512.7±50.80) was more than that of *Cynodon* grass (16540.9±50.80). Larvae recovery per kg DM for different pastures under treatment were in decreasing order as follows: T-2 of paddy (6981.33±50.80, 35.77%), T-2 of *Cynodon* (5545.38±50.80, 33.52%), T-3 of paddy (317378±50.80, 16.26%), and T-3 of *Cynodon* (2218.72±50.80, 13.41%) which showed significant difference (p<0.05) among the treatments. In T-4 (paddy) and T-5 (*Cynodon*), the average number of recovery of larvae was nil implying no significant variation (p>0.05).

**Conclusion:**

This study shown that when N-P-K fertilizers administered at recommended level, significantly reduced larval translation of *H. contortus* minimizing pasture infectivity for the free range grazing animals.

## Introduction

One of the major constraints to the profitable livestock farming is the widespread worm infection worldwide [1]. In livestock, gastrointestinal nematode infections cause a heavy economic loss in terms of ill health, low productivity, morbidity and mortality [2,3]. The situation is challenging in tropics and subtropics due to prevailing of warm and wet climates throughout the year [4,5]. Here, the pasture provides a conducive environment for development, i.e., deposition of eggs, hatching, development of larva, and dissemination of infective larvae of gastrointestinal nematodes to the definitive hosts [6]. Surveys indicated that pasture is the storehouse of parasites mostly *Haemonchus contortus*, *Trichostrongylus* spp., *Oesophagostomum* spp., *Cooperia* spp., and *Mecistocirrus* spp. [7]. Among these, *H. contortus* is the most important gastrointestinal nematode parasite of small ruminants for its wide distribution and high pathogenicity [8]. It inflicts heavy blood loss leading to anemia, anorexia, loss of condition, stunted growth, hypoproteinemia, and even death of animals. In the absence of specific vaccines against many gastrointestinal nematodes due to antigen complexity as well as parasitic immunity, not properly understood, use of anthelmintic has been the main method of their control since long. This has resulted in emergence of anthelmintic resistance in target worms affecting the success rate of reducing the worm burden in the animals [9].

In this context, alternative control methods are being employed and evaluated throughout the world to find a suitable alternative to anthelmintics. In addition, anthelmintics have no distinct role in killing the infective larvae of nematodes present in pasture. Therefore, other chemical agents that can be applied on pasture need to be evaluated for their possible effect, if any, on the pasture larvae.

This experiment was carried out with an aim to study the effect of some commonly used chemical fertilizers on free-living stages of *H. contortus* which is widely prevalent and important from pathogenic point of view. Inadequacy of available literature relating to this type of study was also another motivating factor to undertake this study. Hence, this investigation was attempted to assess whether the N-P-K fertilizers, that are commonly used to fortify the soil to produce more fodder, have any larvicidal effect on the free-living stages of *H. contortus* in pasture.

## Materials and Methods

### Ethical approval

This experiment involved no use of live animals and was conducted in accordance with the guidelines provided by the Institutional Ethical Committee and also complies with the country’s laws.

### Study site

*In vivo* study was conducted for 1 year inside the experimental farm area, Department of Agronomy, Orissa University of Agriculture and Technology (OUAT), Bhubaneswar, Odisha (20° 26’ N, 85° 81’ E, and 34 m above mean sea level). It has a hot humid climate with mean annual rainfall of 1482 mm. The annual mean maximum and minimum temperature of the area were 31.5 and 22.3°C, respectively. The facilities available in the Department of Veterinary Parasitology and other Departments of College of Veterinary Science and Animal Husbandry, Bhubaneswar, Odisha, were utilized to carry out the parasitological studies. Adult *H. contortus* parasites were collected from gastrointestinal tracts of goats slaughtered at local abattoirs of Bhubaneswar, Odisha.

### Collection of H. contortus eggs

Abomasum of freshly slaughtered goats was collected in thermo cool boxes from a local abattoir and brought to the Department of Veterinary Parasitology. The adult female *H. contortus* distinguished from their barber’s pole appearance and other morphological features were recovered from those abomasums cut open in the laboratory [10]. Fertilized eggs were collected from them by dissecting the worms in phosphate buffer saline (PBS). Eggs were counted per ml of suspension in PBS and stored at 15°C for further use [11].

### Formulation of doses of N-P-K fertilizers

The recommended N-P-K dose for paddy cultivation in Odisha is 80-40-40 kg/ha [12], but there exists no such organized cultivation process for *Cynodon* and therefore, no recommended dose is available for it. However, very low doses of N-P-K can be employed for it and keeping this in view; this experimental model was designed by taking the five doses of N-P-K for both the pasture (Table-1). Commercial fertilizers such as urea (N-46%), single super phosphate (SSP) (P-16%), and muriate of potash (MOP) (K-60%) were used here. Based on the doses, the requirement of urea (N), SSP (P), and MOP (K) for area 1 m×1 m were calculated (Table-1), and those were added with tap water (100 ml) to form solution for pasture application.

**Table-1:** Estimation of N-P-K (urea, SSP, MOP) requirement for experimental plot (1 m×1 m).

Name of experimental plot	N-P-K dose (kg/ha)	Urea/plot (g)	SSP/plot (g)	MOP/plot (g)	Water (ml)
T-1: A, F (control)	0-0-0	0	0	0	100
T-2: B, G	10-05-05	2.17	3.13	0.84	100
T-3: C, H	20-10-10	4.34	6.25	1.67	100
T-4: D, I	40-20-20	8.68	12.5	3.34	100
T-5: E, J	80-40-40	17.36	25.00	6.68	100

SSP=Single super phosphate, MOP=Muriate of potash

### Experimental infestation of worm eggs on pasture

Five parasite free experimental pasture plots of 1 m×1 m area each of paddy leaves (15-day-old) and equal number of *Cynodon dactylon* grass were developed (paddy plots: A, B, C, D, and E and *Cynodon* plots: F, G, H, I, and J) inside the experimental farm, Department of Agronomy, OUAT, Bhubaneswar. To avoid intermixing of migrated larvae, bare plots of similar size were made separating the experimental plots, and the surrounding area was fenced to prevent access of stray animals. Each plot was experimentally infested with 10×10^4^ eggs/ml PBS [13] along with the application of the test dilution of fertilizers solution (half amount as spray, another half on the soil) in September with prevailing temperature and relative humidity ranging from 23.8 to 33.7°C and 75-98%, respectively.

### Pasture harvesting

On the 10^th^ day of posttreatment, grass and paddy leaves weighing 100 g each were cut close to the soil of each plot from five places (four corners and center) in morning (7.30-10.00 A.M) when due to phototropism the vertical migration of larvae would have maximum [14,15], collected and stored at 4°C until recovery of larvae [16].

### Recovery, isolation, and identification of larvae from pasture

Soaking method and Baermann technique were adopted for recovery of larvae followed by the slight modification of description of Hansen and Perry, 1994 [17]. Briefly, harvested pasture weighing 100 g each from five places of all plot were kept in a gauze bag (pores size: 1.5 mm×1.5 mm) and was dipped in bucket having 5 L of water and 2.5 ml of TWEEN^®^ 20 detergent (M/S Sigma-Aldrich India, Kolkata, India) at recommended concentration (0.05% v/v). The bags were kept in the bucket such that bottom of the bucket did not touch it. Within 3 h of the process, for proper rinsing, the bags were raised several times inside the bucket.

Then, the bags were left soaking overnight, removed and rinsed with fresh tap water into the bucket for 4 times. The buckets were kept undisturbed for 1 h to form sediment and the supernatant transferred to 500 ml. Then, the sediment was passed through a kitchen tea strainer to remove large grass particles and decanted to 1 L volume. This volume was passed through Baermann apparatus of without screen for 1 h. The sediment (30-40 ml) in the rubber tubing of Baermann apparatus was then collected into a test tube, centrifuged at 2000 rpm for 3 min and kept at 4°C for 1 h. The above supernatant was again concentrated to 10 ml. After proper mixing from 10 ml sample, three 1 ml aliquot samples were taken and examined under compound microscope after iodine staining. All the L_3_ *H. contortus* larvae were identified and counted [10].

### Determination of pasture dry matter (DM) and counting of number of larvae per kg DM

After larval recovery, the DM of each 100 g pasture was determined following standard laboratory procedure [18]. The number of larvae recovered from the pasture of each 100 g was calculated by taking 200 µl from each 1 ml suspension. Then average number of larvae for 200 µl was counted to determine the number of larvae per 10 ml. This determined number of larvae was present in the pasture of 100 g. The estimated number of larvae recovered from 1 kg of dry grass and dry paddy leaves each was calculated using the formula suggested by Krecek *et al.*, 2004 [19].


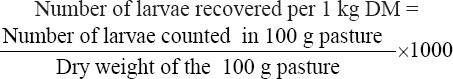


For better understanding L_3_ recovery was expressed as percent compared to L_3_ recovery in control plots considered as 100%.

### Statistical analysis

The data obtained for a number of larvae per kg DM of pasture with respect to different doses of N-P-K fertilizers were analyzed for significance by ANOVA. The means were compared by DMRT using SPSS program version 16.

## Results and Discussion

**Figure-1:**
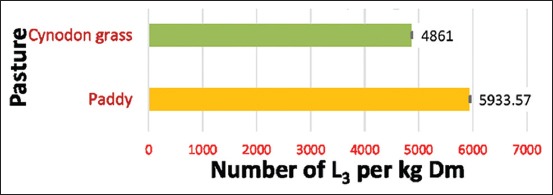
Effect of pastures on mean population of L_3_ recovered per kg dry matter.

The average recovered population of L_3_ of *H. contortus* per kg DM varied significantly (p<0.05) between the paddy leaves (5933.57±22.718) and *Cynodon* grass (4861.00±22.718) (Figure-1). The tallness, more herbage of paddy leaves might help egg and larval establishment due to shade and moisture, so more larval population found as compared to *Cynodon* grass.

However, *Cynodon* is a creeping grass which creeps along the ground, so there may be more exposure to sun ray causing more death of eggs by desiccation. The present findings were akin to the earlier researchers, who observed that the different microclimates produced by the existence of different morphological and biological features among forage species affected survival and establishment of larvae [20,21].

On comparison of different doses and their impacts on different pasture revealed that for control (T-1, 0-0-0 kg/ha N-P-K), the mean L_3_ recovery per kg DM of paddy (19,512.7±50.80, 100%) was more than that of *Cynodon* grass (16,540.9±50.80, 100%), so significant difference (p<0.05) was found between them. Larvae recovery per kg DM for different pastures were in decreasing order: T-2 of paddy (6981.33±50.80, 35.77%), T-2 of *Cynodon* (5545.38±50.80, 33.52%), T-3 of paddy (3173.78±50.80, 16.26%), and T-3 of *Cynodon* (2218.72±50.80, 13.41%) which showed significant variation (p<0.05) among each other (Table-2). So increasing in the N-P-K concentration resulted in significant (p<0.05) inhibition in egg hatching as well as larval development. In T-4 and T-5 of both pasture, the average number of recovery of larvae was nil implied no significant difference (p>0.05) which indicated that when the N-P-K dose approached to 40-20-20 kg/ha or more than that, complete embryonic mortality (acts as larvicidal) occurred. It has been reported that larvae recovery percentage were 19.7, 4.8, 1.2, and 0 at a dose rate of urea 27.17, 54.35, 108.70, and 217.40 kg/ha, respectively [13]. Furthermore, inhibition of egg hatching of *H. contortus* was most effective at 15:15:75 kg/ha N-P-K [22].

**Table-2:** Effect of N-P-K doses on mean population of L_3_ recovered from pastures.

N-P-K in different doses (kg/ha)	L_3_ recovery per kg DM of paddy	Percentage relative L_3_ recovery per kg DM of paddy	L_3_ recovery per kg DM of *Cynodon dactylon*	Percentage relative L_3_ recovery per kg DM of *Cynodon dactylon*
0-0-0 (T-1), control, n=10	19512.7±50.80^a^	100	16540.9±50.80^b^	100
10-05-05 (T-2), n=10	6981.33±50.80^c^	35.77	5545.38±50.80^d^	33.52
20-10-10 (T-3), n=10	3173.78±50.80^e^	16.26	2218.72±50.80^f^	13.41
40-20-20 (T-4), n=10	0±50.80^g^	0	0±50.80^g^	0
80-40-40 (T-5), n=10	0±50.80^g^	0	0±50.80^g^	0
SEM	50.80		50.80	

Means with different superscripts differ significantly (p<0.05) from each other in a row or a column. DM=Dry matter, SEM=Standard error of mean

Production of toxic products such as ammonia [23-25], nitrates, and nitrites [26] from urea caused parasitic larval mortality. When larvae were exposed to fertilizers, change in osmotic pressure triggered water loss from larval cells leading to cell death [26]. Alteration of pH of soil and salinity due to urea or phosphorus fertilizer had adversely affected egg hatching and larval development in *H. contortus* [27] along with in root-knot nematodes *Meloidogyne javanica* and *Meloidogyne incognita* [28]. In other studies, urea might be lethal for juvenile, immature and mature earthworm of *Eisenia foetida*, by the mechanism of skin infiltration [29] and also toxic for filarial vector mosquito, *Culex pipiens* [30]. Some investigators observed that inorganic fertilizer (N-P-K) had potential to suppress cereal cyst nematode, *Heterodera avenae* population [31] as well as root-knot nematode population [32-36].

It was observed that reproduction and infectivity of entomopathogenic nematodes could be checked by N-P-K fertilizer and potassium nitrate application [37]. The inhibition of hatching of *H. contortus* eggs due to fertilizer action might be as a result of disturbance in the secretion or inactiveness of different enzymes present in the eggs for the hatching to occur [38]. Furthermore, some possible mechanism was involved to release various toxic metabolites or certain free radicals from the fertilizers since fertilizers are the potent generator of free radicals which were responsible for blocking the active metabolic process of egg hatching and their subsequent development [39]. Urea, potash, N-P-K fertilizer hindered hatching, larval development and survival of *Aedes aegypti* by alteration of pH, interruption in nervous transmission, conductivity, and total dissolved solid of parasitic developmental stages [40,41].

In addition, the inhibition of development of parasite at higher doses of N-P-K fertilizers observed in this study might be due to their potency at higher doses. Combined dose of N-P-K used, in our study might have acted synergistically for more effective control in the development of larvae. Since N-P-K fertilizer in recommended dose is commonly used in agricultural practices to increase crop yield through enhanced availability of macronutrients, it can be safely recommended for use on pasture. However, available literature does not reveal harmful effects of N-P-K on normal microfauna of the environment.

## Conclusion

It is concluded that administration of N-P-K fertilizers at proper dose will result in significant decrease in worm burden of *H. contortus* in pasture infectivity to grazing animals. The recovery of third stage larvae population of *H. contortus* was found to vary with pasture species.

## Authors’ Contributions

TKR, MRP, BM, and KKS designed the experiment. TKR and MRP carried out the experimental work. TKR, MRP, BM, KKS, MD, AH, and TM were involved in scientific discussion and analysis of the data. TKR, BM, KKS, drafted and revised the manuscript. All authors read and approved the final manuscript.
